# Acetogenic production of 3-Hydroxybutyrate using a native 3-Hydroxybutyryl-CoA Dehydrogenase

**DOI:** 10.3389/fmicb.2022.948369

**Published:** 2022-08-08

**Authors:** Jonathan Lo, Jonathan R. Humphreys, Lauren Magnusson, Benton Wachter, Chris Urban, Skyler D. Hebdon, Wei Xiong, Katherine J. Chou, Pin Ching Maness

**Affiliations:** National Renewable Energy Laboratory, Golden, CO, United States

**Keywords:** metabolic pathway discovery, acetogen, *Clostridium ljungdahlii*, 3-hydroxybutyrate, syngas utilization

## Abstract

3-Hydroxybutyrate (3HB) is a product of interest as it is a precursor to the commercially produced bioplastic polyhydroxybutyrate. It can also serve as a platform for fine chemicals, medicines, and biofuels, making it a value-added product and feedstock. Acetogens non-photosynthetically fix CO_2_ into acetyl-CoA and have been previously engineered to convert acetyl-CoA into 3HB. However, as acetogen metabolism is poorly understood, those engineering efforts have had varying levels of success. 3HB, using acetyl-CoA as a precursor, can be synthesized by a variety of different pathways. Here we systematically compare various pathways to produce 3HB in acetogens and discover a native (*S*)-3-hydroxybutyryl-CoA dehydrogenase, *hbd2*, responsible for endogenous 3HB production. In conjunction with the heterologous thiolase *atoB* and CoA transferase *ctfAB*, *hbd2* overexpression improves yields of 3HB on both sugar and syngas (CO/H_2_/CO_2_), outperforming the other tested pathways. These results uncovered a previously unknown 3HB production pathway, inform data from prior metabolic engineering efforts, and have implications for future physiological and biotechnological anaerobic research.

## Introduction

The Wood Ljungdahl Pathway (WLP), used by acetogens, is considered the most energy efficient natural carbon fixation pathway (Bar-Even et al., 2012). This natural efficiency makes acetogens attractive for biotechnological purposes. Despite the promise of acetogens, the main products natively made are normally acetate and ethanol, which are generally lower value products with limited market sizes. 3-Hydroxybutyrate (3HB) is a chiral bioproduct of interest, with a variety of uses and applications. 3HB can be used for the synthesis of fine chemicals, medicines, biofuels, and bioplastics, especially polyhydroxybutyrate (PHB), which is a highly biodegradable bioplastic (Tokiwa and Ugwu, 2007). 3HB can also be co-polymerized with other biodegradable polymers to extend their use case and improve physical properties (Li et al., 2016; Cywar et al., 2021). With the environmental persistence of petroleum-based plastics and concerns over fossil fuels use and emissions, PHBs offer a potentially environmentally friendly alternative that is both biodegradable and reduces CO_2_ emissions. As a result of these attributes, PHBs are currently industrially produced by a number of companies, representing a growing market and active area of research (Chen, 2009).

As PHB/3HB prices are heavily influenced by substrates costs (i.e., sugar/oils), increased efficiency and use of marginal inexpensive substrates allows for cheaper production and reduced carbon emissions (Lee, 1996). Production of 3HB in acetogens is attractive for several reasons. Acetogens are potentially more carbon efficient at producing 3HB from sugars due to their ability to grow mixotrophically (Jones et al., 2016). Using the WLP, acetogens can recapture CO_2_ from pyruvate decarboxylation to generate 3 acetyl-CoA per glucose, versus the 2 acetyl-CoA normally produced by heterotrophic glycolysis. As such, 3HB production is theoretically 36% more efficient in acetogens on sugar alone and can theoretically result in complete carbon conversion with added H_2_ (Jones et al., 2016). Acetogens can also use syngas to generate acetyl-CoA, a precursor of 3HB biosynthesis, which means 3HB can be generated from carbon emitting sources directly, resulting in a carbon negative product. Recent technoeconomic analysis has shown that PHB from CO_2_ is potentially cost competitive at current prices (Huang et al., 2021). As a result, there is increasing environmental interest in engineering 3HB production in acetogens.

**FIGURE 1:**
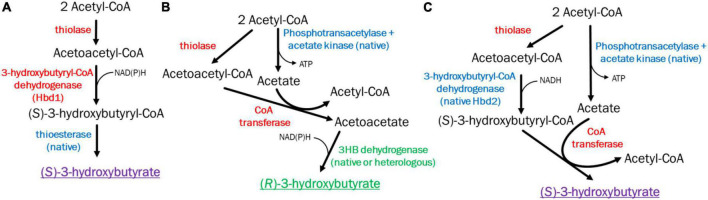
Pathways and enzymes for acetogenic 3HB production. **(A)** (*S*)-3-Hydroxybutyrate production pathway *via* Hbd1. **(B)** (*R*)-3-Hydroxybutyrate production pathway *via* CoA transferase/3Hbdh. **(C)** New (*S*)-3-Hydroxybutyrate production pathway *via* CoA transferase Hbd2. Heterologous enzymes in red, native enzymes in blue, native/heterologous *R*-3HB enzymes in green. *S*-3HB in purple.

In literature, there are two main pathways proposed to generate 3HB in acetogens, with varying levels of success (Figure 1). These two pathways will be referred to by their critical genes as *hbd1* ((*S*)-3-hydroxybutyryl-CoA dehydrogenase) (Figure 1A), and *ctfAB* (CoA transferase)/*3hbdh* (3-hydroxybutyrate dehydrogenase) (Figure 1B) (Jones et al., 2016; Woolston et al., 2018; de Souza Pinto Lemgruber et al., 2019; Flüchter et al., 2019; Karim et al., 2020; Jia et al., 2021). Both pathways require a thiolase to combine two acetyl-CoA molecules into acetoacetyl-CoA, however, from here the pathways diverge. After the formation of acetoacetyl-CoA, Hbd1 reduces acetoacetyl-CoA to (*S*)-3-hydroxybutyryl-CoA which is subsequently converted to 3HB *via* a thioesterase through removal of CoA. Conversely, the *ctfAB/3hbdh* pathway first uses the CtfAB to convert acetoacetyl-CoA to acetoacetate by transferring the CoA to acetate. From here, acetoacetate is then reduced by the 3Hbdh resulting in 3HB (Figure 1). The *hbd1* pathway showed 3HB high titers from syngas (Karim et al., 2020), while *ctfAB*/*3hbdh*, relatively understudied, showed some ability to produce 3HB (Jones et al., 2016; Flüchter et al., 2019; Jia et al., 2021). The (R)-3-hydroxybutyryl-CoA dehydrogenase gene *phaB*, from *Cupravidus necator*, has also been utilized but showed poor 3HB production compared to the other pathways (Woolston et al., 2018; Flüchter et al., 2019). In this study, we investigated these promising 3HB production pathways in the acetogen *Clostridium ljungdahlii*, to compare their ability to make 3HB and explore methods to improve 3HB production.

Different 3HB pathways have different implications for productivity, specificity, cellular metabolism, ATP productivity, and redox (Bertsch and Müller, 2015; Woolston et al., 2018; Emerson et al., 2019). For instance, while *hbd1* appears promising with regards to titer, its performance on syngas was inconsistent, with high and low 3HB production reported (Woolston et al., 2018; Karim et al., 2020). This may be because pathways using *hbd1* enzymes do not produce ATP *via* substrate level phosphorylation (Figure 1A). As cell metabolism requires surplus ATP for growth and maintenance, acetate production may be required for ATP production. Thus, acetate production may “subsidize” 3HB production. In this pathway acetate is a terminal product and diverts acetyl-CoA from 3HB (Emerson et al., 2019).

In contrast, the *ctfAB/3hbdh* pathway relies on a CoA transferase of acetoacetyl-CoA to acetate, generating acetyl-CoA and acetoacetate, which is then reduced by 3HB dehydrogenase (3HBDH) to 3HB. The *ctfAB/3hbdh* pathway uses acetate as an intermediate and regenerates acetyl-CoA (Figure 1B). As a result, this pathway has a better ATP yield, which is important as growth on syngas is ATP limited (Bertsch and Müller, 2015; Emerson et al., 2019). Data on the *ctfAB/3hbdh* pathway showed promise but had inconsistent results. Jones et al suggested that acetoacetate can be natively converted to 3HB, as they saw significant levels of 3HB from their acetone generating strain which contained all genes required for the *ctfAB/3hbdh* pathway without *3hbdh* itself (Jones et al., 2016). Data from Flüchter et al. (2019) suggests the *ctfAB/3hbdh* was highly active in *Clostridium coskatii* under heterotrophic conditions, but not autotrophically. This pathway was also tested in *C. ljungdahlii* but did not function under their conditions. While not optimizing for 3HB, Jia et al. (2021), also showed good titers (3 g/L) in *C. ljungdahlii* from this pathway, but low carbon yields (∼7%).

In investigating these pathways, we uncovered a pathway that performed substantially better in *C. ljungdahlii*, relying on *ctfAB* and an endogenous *hbd2*, referred to as *ctfAB/hbd2* (Figure 1C). Annotated as (*S*)-3-hydroxybutyryl-CoA dehydrogenase (EC 1.1.1.57), little is known about *hbd2*. *Hbd2* is an NADH specific enzyme, however, the functions and applications of this enzyme are not well known (Seedorf et al., 2008; Wang et al., 2010; Karim and Jewett, 2016). As far as we know, this work is the first time *hbd2* has been validated for *in vivo* targeted product formation. We chose to investigate *hbd2* further to both improve 3HB titers from syngas and expand our knowledge of the function and potential applications of this enzyme.

## Materials and methods

### Microbial strains and media composition

*Clostridium ljungdahlii* DSM 13528 and *Clostridium kluyveri* DSM 555 were from The Leibniz Institute DSMZ (Germany). *Clostridium acetobutylicum* ATCC 824 was from American Type Culture Collection (Manassas, VA, United States). *C. ljungdahlii* growth manipulations were based on previously reported techniques (Lo et al., 2020). Routine growth was performed at 37°C in modified YTF media (10 g/L yeast extract, 16 g/L Bacto tryptone, 4 g/L sodium chloride, 5 g/L fructose, 0.5 g/L cysteine, pH 6). YT media was the same as previous, omitting fructose as a carbon source. Bacterial manipulations were performed in a COY chamber (COY lab, Grass Lake, MI, United States) maintained anaerobic *via* palladium catalyst with 95% N_2_ and 5% H_2_ from Airgas (Randor, PA, United States). In general, chemical reagents were purchased from Sigma-Aldrich (St. Louis, MO, United States) or Thermo Fisher (Waltham, MA, United States), unless otherwise indicated.

### Molecular techniques

Standard molecular cloning and PCR techniques were used with enzymes from New England Biolabs (Ipswich, MA, United States). Routine PCR was performed using Phusion polymerase. For routine cloning and plasmid propagation, *Escherichia coli* strain NEB 10-Beta was utilized from New England Biolabs. The 1 kb Opti-DNA Marker ladder was from Applied Biological Materials (Vancouver, Canada). Primers and *C. ljungdahlii* optimized genes were generated from IDT (Coralville, IA, United States). Codon optimized genes *atoB* from *E. coli*, 3HBDH from *Rhodobacter sphaeroides*, the 3HBDH from *Clostridium difficile* were generated from the IDT algorithm using the *Clostridium acetobutylicum* option, which has a similar codon usage as *C. ljungdahlii*. Exact codon optimized sequences and primers are described in the Supplementary Materials. The pMTL80000 plasmids were from Chain Biotech (Nottingham, United Kingdom). Plasmids were generated using Gibson assembly from NEB. Confirmation of plasmids was performed by whole plasmid sequencing from the MGH DNA Core Facility (Cambridge, MA, United States).

Preparation of electrocompetent cells and transformation was performed based on previously reported protocols (Leang et al., 2013). Briefly, cells were grown overnight on YTF containing 40 mM DL-Threonine to an OD of 0.2–0.7, harvested, then washed with ice cold SMP buffer (270 mM sucrose, 1 mM MgCl_2_, 7 mM sodium phosphate, pH 6), then resuspended in SMP buffer with 10% (dimethyl sulfoxide) DMSO and frozen at –80°C until transformed. Cells mixed with 2–10 μg of DNA in a 1 mm cuvette, then transformed using a Bio-Rad Gene Pulser Xcell Electroporator (Hercules, CA, United States) with the following conditions: 625 kV, resistance at 600 Ω, capacitance of 25 μF. Cells were recovered overnight in YTF and plated the next day embedded in molten YTF 1.5% agar with 10 μg/mL thiamphenicol. Colonies appeared after 3 days. For generating the “3HB integration strain”, cells were grown in liquid YTF with thiamphenicol and 500 μg/mL 5-Fluoroorotic Acid (5FOA) based on previously reported protocols (Olson and Lynd, 2012; Minton et al., 2016). Cells were then single colony plated in YTF agar with thiamphenicol and 5FOA, picked, and colony screened using PCR. To cure the thiamphenicol resistant plasmid, PCR confirmed colonies were then passaged in YTF without thiamphenicol until thiamphenicol sensitivity was restored.

### Analytical techniques

Liquid fermentation products were processed *via* previously described methods (Marcano-Velazquez et al., 2019). Briefly, samples were collected and filtered using Corning Costar Spin-X 0.45 μm (Corning, NY, United States) and routinely measured *via* HPLC, on a 1200 series Agilent (Santa Clara, CA, United States) Aminex HPX-87H column at 55°C with a 4 mM H_2_SO_4_ mobile phase. Enzymatic determination of *R*-3-Hydroxybutyrate was performed on HPLC filtered samples using the “D-3-Hydroxybutyric Acid (β-Hydroxybutyrate) Assay Kit” from Megazyme (Ireland), using the manufacturer’s instructions for the 96 well plate-based assay on a Tecan infinite M200 pro plate reader (Tecan Life Sciences, Switzerland). (*R*)-3-Hydroxybutyric acid and (*S*)-3-Hydroxybutyric acid were purchased from Sigma-Aldrich and used as standards from a 1 to 20 mM concentration. Optical density was measured using a Nanodrop (Thermofisher Scientific, Waltham, MA) at 600 nm. Carbon distribution was determined using only measured liquid components (i.e., Acetate, Ethanol, 3-Hydroxybutyrate, Fructose), measuring the mM and multiplying by the number of carbons in each product.

### Growth conditions

Heterotrophic growth of the strains was carried out in 15 mL Falcon tubes (Fisher scientific) using a 4 mL YTF medium with/without the addition of 10 μg/mL thiamphenicol at 37°C. Cells from an overnight seed culture were added in a 1:5 ratio (1 mL culture into 4 mL YTF) and left for 3 days before sampling. Autotrophic growth with CO, CO_2_, and H_2_ was carried out using 250 mL Duran Pressure Plus bottles (DWK Life Sciences, USA) containing 50 mL YT medium (YTF without fructose). 10 mL of an overnight culture was added to the bottles. Bottles were sealed and aseptically flushed with a CO, CO_2_, and H_2_ mixture (70%/20%/10% CO/CO_2_/H_2_) for 3 min. The same gas mixture was then added to 6 PSI of pressure within the bottles. YT within the bottles was supplemented with 10 μg/mL thiamphenicol for growth with plasmid bearing strains. Bottle growth was carried out at 37°C with 200 RPM shaking.

### Bioreactor conditions

Autotrophic bioreactor growth was carried out using an Electrolab 2L bioreactor, containing 1.70 L YT with 10 μg/mL thiamphenicol. 300 mL of an autotrophic seed culture was added, and growth was carried out with a CO, CO_2_, and H_2_ mixture (70%/20%/10% CO/CO_2_/H_2_) at a flow rate of 300 standard cubic centimeter per min (sccm) with a fine steel diffusion stone. The pH was maintained at 5.2 using 3 M NaOH and the temperature was kept at 37°C using a heating wrap. Initial stirring was at 300 RPM which was increased to 500 RPM, followed by 900 RPM once cells began to grow based on OD_600_. OD_600_ and HPLC samples were taken daily.

### Enzyme assays

Enzyme assays were performed based on previously reported procedures (Madan et al., 1973; Lo et al., 2020). Briefly, 50 mL of *E. coli* cells expressing *C. kluyveri hbd1*, *C. ljungdahlii hbd2*, and control vector pMTL83151 were harvested at mid-log phase and kept at –80°C until the day of enzyme assays. The lysis was performed using a bead-beating method. The 3-Hydroxybutyryl-CoA dehydrogenase assay was performed under the following conditions: 100 mM potassium phosphate buffer (pH 6.5), 25 mM potassium citrate, 75 μM NAD(P)H, and 125 μM acetoacetyl-CoA in 200 μL 96-well plate with a BioTek Synergy Neo2 plate reader (BioTek Instruments, United States) at 6 second intervals for 10 min. Oxidation of NAD(P)H at 340nm was used to follow enzyme activity, which is reported as μmol min^–1^ mg^–1^. To control for non-Hbd2 activity, we subtracted activity from *E. coli* cell free extract with pMTL83151, which was low (<0.01). Both *hbd1* and *hbd2 E. coli* cell free extracts showed low activity without acetoacetyl-CoA (<0.01). Cell free extract protein was measured using a Bradford assay.

## Results

### 3HB production *via* an acetoacetate intermediate

3HB production *via* acetoacetate is a straightforward pathway, requiring three steps: thiolase (Thl) to condense two acetyl-CoA to acetoacetyl-CoA, acetate:acetoacetyl CoA transferase (CtfAB) to transfer the CoA from acetoacetate-CoA to acetate, generating acetyl-CoA and acetoacetate, and 3Hbdh, which catalyzes the reduction of acetoacetate to 3HB (Figure 1B). We were initially interested in the relatively understudied *ctfAB/3hbdh* pathway due to previous literature showing substantial unintended 3HB production during *C. ljungdahlii* acetone/isopropanol production *via* Thl and CtfAB. First, we wanted to determine whether *C. ljungdahlii* contains an effective and native enzyme to efficiently reduce exogenous acetoacetate to 3HB as previously suggested (Jones et al., 2016; Jia et al., 2021). A similar study had been done by spiking in acetone, whereby a native reductase efficiently converted +90% acetone to isopropanol (Köpke et al., 2014), making it unnecessary to express a separate acetone reductase.

We therefore added 15 mM of acetoacetate to a growing culture of *C. ljungdahlii* to test endogenous reduction of acetoacetate. We measured ∼5 mM 3HB, demonstrating that acetoacetate can be natively reduced to 3HB. However, as only ∼5 mM of 3HB was detected, this suggests two thirds of the acetoacetate had been lost, and native acetoacetate conversion to 3HB is low.

Since it appears that acetoacetate reduction could be a limiting factor, we wanted to test various 3Hbdhs that could improve acetoacetate reduction. For the thiolase reaction, we chose the thiolase *atoB* from *E. coli* which showed good performance in *C. acetobutylicum* for butanol production (Nguyen et al., 2018). For the CoA transferase, we chose the *ctfAB* from *C. acetobutylicum* since this enzyme is well characterized and previously showed good functionality in *C. ljungdahlii* (Jones et al., 2016). We then tested putative 3HBDHs previously from literature: one from *C. difficile* (CDIF630_02933, native sequence and a codon optimized version), one from *Rhodobacter sphaeroides* (Rsph17025_1507), and one from *C. ljungdahlii* (CLJU_c23220) (Jones et al., 2016; Flüchter et al., 2019). We also note that the native *C. ljungdahlii* gene we overexpressed is not the gene responsible for 3HBDH activity in a recently published paper (Jia et al., 2021). Nevertheless, the overexpression of CLJU_c23220 serves as a “non-*3hbdh*” overexpression control.

**FIGURE 2:**
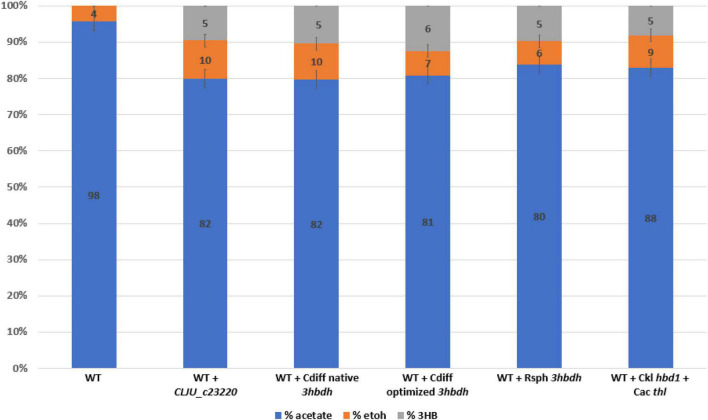
Heterotrophic 3HB production by *Clostridium ljungdahlii* strains expressing different 3HB pathways. Carbon distribution of fermentation products is indicated by the bar chart %, while the number label is the mM amount after 5 days of growth. In addition to the thiolase (*thl*) and CoA transferase, four genes were expressed on separate replicating plasmids. A codon optimized 3-Hydroxybutyrate dehydrogenase (*3hbdh*) from *R. sphaeroides* (Rsph), from *C. difficile* (Cdiff), native and codon optimized, and Clju_C23220 were expressed. Note that the *C. ljungdahlii* gene expressed is not a true 3HB Dehydrogenase (see text). A separate plasmid containing only *C. kluyveri hbd1* and *C. acetobutyulicum* thiolase (*thl*) was also expressed. Error bars show standard error of the mean (SEM) for 3 biological replicates.

We also wanted to compare the *hbd1* pathway to our *ctfAB/3hbdh* pathway due to a report of high 3HB titers in *C. autoethanogenum* (Karim et al., 2020). The *hbd1* pathway uses the *C. acetobutylicum* thiolase (Cac *thl*) and *hbd1* from *Clostridium kluyveri* (Figure 1A). Expression of the pathways on each tested plasmid was driven by a *C. ljungdahlii* ferredoxin promoter (P*fdx*) to allow for equal comparison. Plasmid bearing strains were grown in YTF medium containing fructose to observe heterotrophic production of 3HB. Final 3HB were determined after 3 days of growth (Figure 2).

Results showed significant heterotrophic 3HB production via these both pathways. The transformed constructs demonstrated 5–6 mM of 3HB, which was comparable to the *hbd1* based pathway (Figure 2). This is consistent with expectations reported by Jones et al and Flüchter et al that acetogenic heterotrophic 3HB production through this pathway is possible. Surprisingly, while Flüchter et al., 2019 specifically tried to express this pathway in both *Clostridium coskatii* and *C. ljungdahlii*, they were only able to demonstrate 3HB production in *C. coskatii*, as they did not detect 3HB production in *C. ljungdahlii*. Nevertheless, our data suggested that this pathway is in fact functional for 3HB production in *C. ljungdahlii*. Interestingly, explicit *3hbdh* expression had only a marginal improvement on 3HB titers (2 mM difference between ‘Clj’ and ‘Cdiff’), despite our data showing native 3HBDH activity was poor.

### Creation of an “integrated 3HB strain”

**FIGURE 3:**
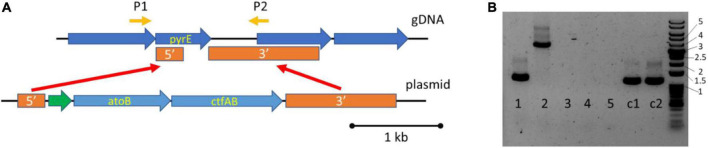
Integration of atoB and ctfAB into the pyrE locus of *Clostridium ljungdahlii* to generate the “integrated 3HB strain.” **(A)** Genome integration of atoB and ctfAB into pyrE locus, driven by P_*fdx*_ in green. P1 & P2 are primer binding sites for screening genomic pyrE locus. **(B)** PCR confirmation of integrated atoB and ctfAB using primers P1/P2 to amplify genomic locus. Lanes 1–5 contain screened 5FOA colonies, C1 and C2 are control gDNA from wild type DNA. A successful integration generates a size of 3.8 kb (Lane 2), whereas the wild type size is 1.2 kb (Lane 1, C1, and C2). The numbers next to the DNA ladder indicate kb size.

**FIGURE 4:**
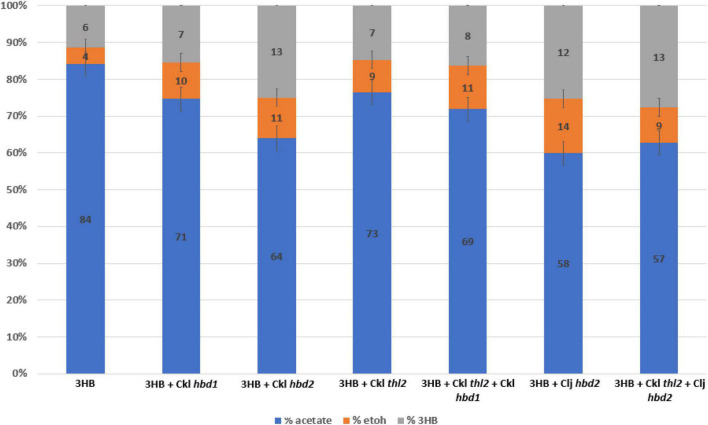
Heterotrophic fermentation product profile of *Clostridium ljungdahlii* strains expressing *hbd1* vs. *hbd2* in the 3HB strain background. Carbon distribution of fermentation products is indicated by the bar chart%, while the number label is the mM amount after 5 days of growth. The “integrated 3HB strain” contains the *atoB* from *Escherichia coli* and CoA transferase from *C. acetobutylicum* integrated into the genome. Replicating plasmids expressing a combination of thiolase, *hbd1*, and *hbd2* were transformed into the integrated 3HB strain and measured for 3HB production. The expression of *hbd2* results in superior 3HB production. Ckl for *C. kluyveri*, Clj for *C. ljungdahlii*. Carbon distribution to 3HB out of the total products is given as a percentage above bars. Error bars show standard error of the mean (SEM) for 3 biological replicates.

To improve 3HB titers, we planned on coexpressing the *ctfAB/3hbdh* and *hbd1* based pathways. We decided to integrate *atoB* and *ctfAB* into the *pyrE* locus to serve as base strain to allow us to test strategies for improving 3HB production with different expression constructs. Using 5-Fluoroorotic Acid (5FOA), homologous recombination can be used to place genes of interest into the *pyrE* locus, as *pyrE*^+^ cells are sensitive to 5FOA (Figure 3) (Minton et al., 2016). The genes *atoB* and *ctfAB* were successfully integrated into the genome, generating a strain that was 5FOA resistant. To cure the replicating plasmid, we subsequently passaged the strain on non-selective YTF media. We then isolated colonies that had become thiamphenicol sensitive, allowing for further transformations. PCR confirmation of integration was also carried out (Figure 3B). This is referred to as the “integrated 3HB strain” and was confirmed to make 3HB (Figure 4).

### Identification of a native *hbd2* for improving 3HB production

Introduction of a CoA-transferase based acetone/isopropanol pathway noted significant 3HB production, even though acetoacetate should be rapidly decarboxylated to acetone in those strains (Jones et al., 2016; Jia et al., 2021). Our experiment indicated that native acetoacetate conversion to 3HB was not robust, with only a third of the acetoacetate converted to 3HB. In light of this evidence, it seems suspicious that previously reported 3HB was derived from acetoacetate, given poor 3HBDH activity and robust decarboxylation to acetone. Explicit *3hbdh* expression did improve 3HB titers (Figure 2), but not as much as we were expecting. While we were prospecting genes for overexpression, we noticed an annotated *hbd2* in *C. ljungdahlii* and wondered whether it could be partially responsible for 3HB production. Transcriptomics evidence shows *hbd2* (CLJU_c37300) is moderately expressed in *C. ljungdahlii* (∼300 FPKM), and we speculated this native gene could play a role in 3HB production (Nagarajan et al., 2013). In contrast, *hbd1* (CLJU_c23560) was not expressed (>1 FPKM) and therefore not pursued.

We generated a *hbd2* expression construct similar to the ones expressed in *C. autoethanogenum* (Figure 1A) (Karim et al., 2020). The constructs in Karim et al. only expressed thiolases and 3-hydroxybutyryl-CoA dehydrogenases but were very effective at generating 3HB in *C. autoethanogenum.* Our *hbd2* construct used the ferredoxin promoter (P*_*fdx*_*) to express a *C. kluyveri* thiolase (*thl2*) and the *hbd2* from *C. ljungdahlii*. Since we were looking for further 3HB production enhancement, we first transformed the *thl2 hbd2* construct into our “integrated 3HB strain.” Surprisingly, we found a 2.5-fold enhancement of 3HB production, from a 3HB carbon-yield of 11% (∼5 mM) to around 25% (∼12 mM) (Figure 4).

We wondered whether *C. kluyveri thl2* and *C. ljungdahlii hbd2* could be efficient alone at making 3HB as was seen in Karim et al, so we transformed them into the wild-type background without the integrated thiolase and CoA transferase. Interestingly, we saw no 3HB production in this strain (data not shown), suggesting that *ctfAB* is a critical gene, presumably to remove the CoA group from the 3HB-CoA generated by *hbd2*. A literature search showed a similar 3HB pathway in *E. coli*, using CoA transferase in conjunction with PhaB as the acetoacetyl-CoA reductase, where the CoA transferase was essential (Matsumoto et al., 2013). To compare *C. kluyveri hbd1*, we also transformed *hbd1* into the “3HB integrated strain,” which showed improved 3HB production to 7.5 mM (∼2 mM over the 3HB parent) but was far inferior to the *hbd2* strains (∼12 mM total). *C. kluyveri* also contains a *hbd2* (CKL_2795), which we transformed as well, and performed similarly to the *C. ljungdahlii hbd2* in 3HB titer (∼12 mM). This indicates that the *hbd2* gene itself is important for increasing 3HB flux and is superior to the *hbd1* gene in our system. In contrast, the plasmid expressing only *thl2* in the “integrated 3HB strain” slightly improved 3HB to 7.5 mM, showing thiolase expression was not a major factor for 3HB production.

The *C. kluyveri* cofactor specificities of Hbd1 and Hbd2 are known from previous work (NADPH for Hbd1 and NADH for Hbd2) (Madan et al., 1973; Wang et al., 2010), but unknown for *C. ljungdahlii* Hbd2. Using cell free extract of *E. coli* expressing *C. ljungdahlii hbd2*, we were able to measure a specific activity of 0.07 μmol min^–1^ mg^–1^, and we confirmed NADH specificity and acetoacetyl-CoA-dependence. *C. kluyveri hbd1 E. coli* extracts were used as an NADPH-specific control and measured a specific activity of 0.22 μmol min^–1^ mg^–1^. We attempted to determine K_*m*_ values for the *C. ljungdahlii* Hbd2 enzyme by varying either the NADH or the acetoacetyl-CoA concentrations. However, during this assay we noticed decreasing Hbd2 enzyme activity over several hours, inconsistent with the expected substrate loading conditions. We repeated the standard conditions of 75 μM NAD(P)H and 125 μM acetoacetyl-CoA with the *hbd2* cell-free extracts, and we found markedly worse Hbd2 activity. In contrast, Hbd1 activity appeared to be intact, indicating that Hbd2 loses activity over time, even when kept on ice. We then lysed *C. ljungdahlii* cell-free extracts expressing *hbd2* and found similar inconsistent activity results, suggesting that this was an Hbd2 related phenomena.

### Stereoisomer of *hbd2* 3HB production and relative contribution of different pathways

**FIGURE 5:**
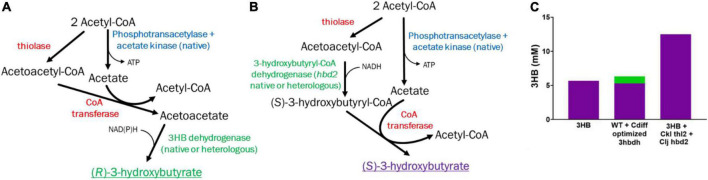
(*R*) vs. (*S*) 3HB Pathways and quantification. **(A)** The *R*-3HB pathway **(B)** The *S*-3HB pathway. Red indicates heterologous genes, blue native genes, green native or heterologous. **(C)** *R* (in green) vs. *S* (in purple) 3HB distribution from heterotrophic fermentation samples using HPLC and enzymatic analysis.

The stereoisomer of 3HB is important to determine, as the stereoisomer can determine its suitability for specific use cases and change bioplastics thermal/mechanical properties. Bioplastics physical properties can be driven and altered by the stereoisomer of the monomers (Cywar et al., 2021). Importantly, we wanted to note the relative contribution of the pathways in 3HB production, which can be determined by measuring the *R* or *S* form of 3HB. In the “integrated 3HB strain”, there are two possible pathways functioning to produce 3HB: the *ctfAB/3hbdh* and *ctfAB/hbd2* pathway. *3hbdh* pathway produces the *R* form (Figure 5A), while *hbd2* produces the *S* form (Figure 5B), and thus their relative contribution can be determined. While we quantified total 3HB via HPLC, we could not resolve the composition of 3HB stereoisomers, so we needed an alternative method.

To confirm that *ctfAB*/*hbd2* was the main cause of 3HB production, versus the *ctfAB/3hbdh* endogenous pathway, we used a commercial *R*-3HB enzyme assay kit (See Materials and Methods) to detect *R*-3HB from the heterotrophic samples from Figures 2, 4. We tested three strains: the “integrated 3HB strain”, 3HB + *Ckl thl2* + *Clj hbd2*, and the wild type with *ctfAB/3hbdh* optimized from *C. difficile*. If the *ctfAB/3hbdh* pathway was the main pathway, we should get a detection from the enzymatic assay as it is specific for *R*-3HB, while *S*-3HB will not give a response. We did not detect any *R*-3HB in any of the “integrated 3HB strains” without *3hbdh* overexpression <0.2 mM). In the wild type harboring the *ctfAB/3hbdh* with the optimized *3hbdh* from *Clostridium difficile*, we detected around 1 mM *R*-3HB, which is around the magnitude of increase viewed *in vivo* with the added *3hbdh* on the plasmid (Figure 5C). This shows that the 3HB produced is mainly *S*-3HB, via the *ctfAB/hbd2* pathway, and not *R*-3HB via acetoacetate reduction with 3HBDH. As a control, we added known amounts of *R* and *S*-3HB and confirmed only *R*-3HB is detected via this assay.

### Autotrophic fermentation of select engineered strains

**FIGURE 6:**
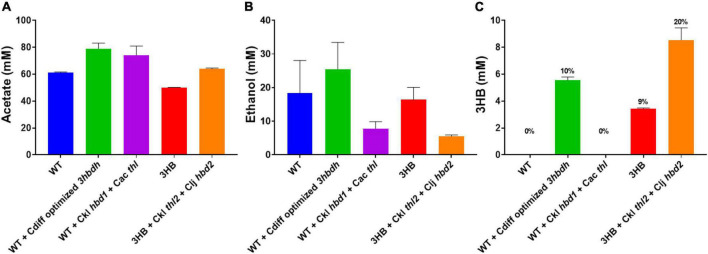
Autotrophic 3HB production in bottle fermentations. Strains were grown on syngas in 250 mL pressure bottles with 70% CO, 10% H_2_, and 20% CO_2_. **(A)** Acetate production, **(B)** ethanol production, and **(C)** 3HB production. Carbon distribution to 3HB out of the total products is given as a percentage above bars. Error bars show standard error of the mean (SEM) for 3 biological replicates.

Next, we tested the performance of select strains growing on syngas. Previous experiments with acetogens showed drastically different 3HB production on syngas versus on sugar. For instance, in two previous works, high *C. ljungdahlii* 3HB titers (∼20 mM) were generated on fructose. However, that did not translate to autotrophic 3HB production (>1 mM) (Woolston et al., 2018; Flüchter et al., 2019). Thus, we took promising heterotrophic strains and tested their autotrophic performance in bottles (Figure 6). Each strain was grown using a syngas mix of 70% CO, 20% CO_2_, and 10% H_2_ and product titers were analyzed after growth and gas consumption ceased. The *hbd1* based pathway did not generate detectable 3HB. The “integrated 3HB strain” generated a small amount of 3HB, around 4 mM. The *atoB, ctfAB*, and *3hbdh* strain generated the next most at ∼6 mM, while the “integrated 3HB strain” +*thl2*+ *hbd2* strain generated the most at over 8 mM 3HB.

### Syngas bioreactor 3HB production

**FIGURE 7:**
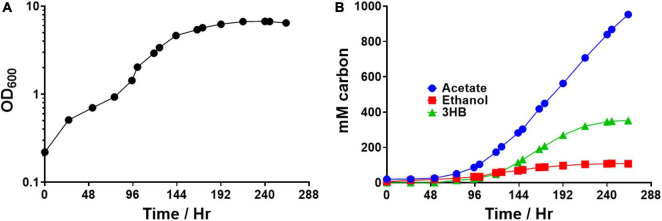
Autotrophic bioreactor fermentation product profile of *Clostridium ljungdahlii* integrated 3HB strain expressing additional *thl* and *hbd2*. The strain contains the *atoB* thiolase from *Escherichia coli* and CoA transferase from *C. acetobutylicum* integrated into the genome with an additional *thl2* and *hbd2* overexpressed on a replicating plasmid. **(A)** Optical density over time **(B)** Fermentation product profile over time in mM carbon.

We then tested the autotrophic performance of the 3HB + *thl2*+ *hbd2* strain during bioreactor growth. We ran the strain under autotrophic conditions in a 2 L bioreactor, feeding 300 sccm of 70% CO, 10% H_2_, 20% CO_2_ maintained at pH 5.2 (Figure 7). The gas fermentation was run for 263 h after which growth ceased. By the end, the strain had produced 476 mM acetate, 53 mM ethanol, and 88 mM 3HB, with a carbon distribution of 67%, 8%, and 25%, respectively. The highest 24-h 3HB productivity rate (that is, the max 3HB rate over a 24-h period) was 0.083 g/L/hr. This shows that the improvement in 3HB production also translates to autotrophic fermentations on syngas. It is also higher than the previously highest titer (28 mM) and yield (∼7%) reported in *C. ljungdahlii* from syngas fed-batch fermentation, which had focused on enhancing 3HBDH to improve 3HB titers (Jia et al., 2021).

## Discussion

Our data shows that among the different 3HB pathways, *ctfAB*/*hbd2* expression outperformed other pathways we tested in *C. ljungdahlii*. Furthermore, *ctfAB/hbd2* was probably responsible for 3HB detection in previous reports targeting isopropanol/acetone production (Jones et al., 2016; Jia et al., 2021). Despite *C. ljungdahlii* native 3HBDH activity, acetoacetate conversion is poor, and seems unlikely to outcompete acetoacetate decarboxylation to acetone in those strains. Rather, our data indicates that the native *hbd2* was likely responsible for the 3HB in those strains, and targeted overexpression of *hbd2* can enhance the yield of 3HB over other tested pathways.

What explains the improved 3HB production by this pathway? The most likely answer lies in Hbd2 (*S*)-3-hydroxybutyryl-CoA dehydrogenase. It has been previously shown in *in vitro* and *in vivo* systems that Hbd activity is key to driving high titers of both butanol and 3HB production, especially since thiolase condensation of acetyl-CoA is an endergonic reaction and downstream reactions are needed to “pull” the reaction forward (Karim and Jewett, 2016; Nguyen et al., 2018; Woolston et al., 2018; Karim et al., 2020). Interestingly, *C. kluyveri hbd2* was tested for 3HB production and was found to underperform in a cell-free system compared to *hbd1* (Karim et al., 2020). The cell-free system used in these experiments did not have an explicit CoA-transferase, which may explain its *in vitro* underperformance. The *hbd2* from *C. beijerinckii* was used *in vitro* to generate butanol, with superior performance versus *hbd1*, but as far as we know this was the only application examined for *hbd2* and was not performed *in vivo* (Karim and Jewett, 2016). The two *in vitro* studies provided conflicting evidence of the effectiveness of *hbd2* vs. *hbd1* and are the only biotechnical applications of *hbd2* reported in literature.

Another explanation could be the presence of CoA-transferase in the 3HB strain. We note that previous work in acetogens found good 3HB production on heterotrophic conditions but poor results on autotrophic conditions (Woolston et al., 2018; Flüchter et al., 2019). It has been shown that acetyl-CoA levels can drastically change depending on heterotrophic versus autotrophic growth (Emerson et al., 2019), so autotrophic flux towards 3HB may be improved by regenerating acetyl-CoA for (*S*)-3-hydroxybutyryl-CoA dehydrogenase activity. In *E. coli*, Matsumoto et al., 2013 elucidated a CoA-transferase dependent 3HB pathway, where 3HB-CoA (generated by PhaB from *Cupriavidus necator*) would transfer the CoA to acetate, generating 3HB and acetyl-CoA. This pathway proved surprisingly efficient, generating a 3HB titer of 1 g/L. Relevant to acetogens, the 3HB titer was improved to 5.2 g/L with high acetate concentrations.

Little is known about *hbd2* even though several *Clostridia* possess the gene. A BLAST search with *C. ljungdahlii* and *C. kluyveri hbd2* indicates high identity to proteins in several *Clostridia* of scientific interest. *Clostridium beijerinckii, Clostridium botulinum, Clostridium coskatii, Clostridium carboxidivorans* and *C. autoethanogenum* generated high identity (>70%) hits with *hbd2*. Interestingly, *C. acetobutylicum* only has an *hbd1*, and not *hbd2*. In contrast, the identities between *hbd2* vs. *hbd1* from *C. kluyveri* have ∼40% amino acid identity to each other, suggesting that these enzymes are phylogenetically different. Butyryl-CoA synthesis genes often form an operon with *hbd1*, indicating a clear role in butyrate/butanol synthesis (Boynton et al., 1996; Hu et al., 2016). In contrast, as far as we can tell, in both *C. kluyveri* and *C. ljungdahlii, hbd2* are co-localized to genes unrelated to butyrate/butanol synthesis.

The *C. kluyveri* Hbd2 is NADH-linked, but its functionality is unknown. It is speculated to be important for redox balancing and chain elongation in *C. kluyveri*, where *hbd1* is notably NADPH-linked (Wang et al., 2010). Almost nothing is known about *hbd2* in *C. ljungdahlii*. We confirmed that *C. ljungdahlii* Hbd2 is NADH specific, like *C. kluyveri* Hbd2, but its function remains a mystery. *C. ljungdahlii* does not natively produce 3HB, PHB, butyrate, or butanol, and its genomic context doesn’t appear to contain any obvious clues. Interestingly, it is moderately expressed in both heterotrophic and autotrophic conditions (FPKM 339 and 291, respectively) (Nagarajan et al., 2013), suggesting it could have an undetermined metabolic role.

This work put into context several interesting pieces of data, especially for the production of 3HB. *C. ljunghdahlii* has a number of functional genes that can natively catalyze 3HB production. It naturally converts acetoacetate to 3HB and has a highly functional Hbd2 that converts acetoacetyl-CoA to 3HB-CoA. 3HB has been produced in *C. ljungdahlii* and related acetogens, and although it was assumed that the heterologous expression of pathway components was responsible for 3HB production, native enzymes may also be playing a role in 3HB production. Published work expressing *ctfAB* in *C. ljungdahlii* assumed that 3HB was derived from acetoacetate reduction, but this 3HB could be from Hbd2 reducing acetoacetyl-CoA. One way to determine the relative contribution is through determination of the R vs. S stereoisomer via enzymatic assay analysis, which is a cheaper alternative than purchasing a chiral column. Furthermore, as different pathways have been expressed to produce 3HB in acetogens, 3HB titers could be increased by combining different pathway strategies into a single organism. We show that both *hbd2* and *3hbdh* can be functional, as we did detect both *S* and *R*-3HB, but the *3hbdh* contribution was low compared to *hbd2.*

Finally, this *ctfAB*/*hbd2* pathway may have other advantages vs. previously described pathways. In our experimental conditions, *hbd2* expression appears to be superior to *hbd1* and *3hbdh*. As a native acetogen/*Clostridia* derived enzyme, the Hbd2 may function better in its native host than heterologous enzymes. For instance, *phaB* has been tested in *C. ljungdahlii* with poor results, possibly due to compromised expression, despite good results in *E. coli* (Matsumoto et al., 2013; Woolston et al., 2018; Flüchter et al., 2019). Furthermore, the CoA transferase from 3-hydroxybutyryl-CoA to acetate regenerates acetyl-CoA, which may be important for acetyl-CoA concentrations and addressing ATP limitation when growing on H_2_/CO_2_/CO. For instance, previously described Hbd1-based 3HB production does not involve substrate-level phosphorylation, whereas the *ctfAB*/*hbd2* described pathway would.

While we have shown good production of 3HB with our new pathway using *ctfAB*/*hbd2*, we believe there is the opportunity for much further optimization and application. We have only tested only *ctfAB* from *C. acetobutylicum* and two *hbd2*, but BLAST screening shows a multitude of genes that could be tested. Greater 3HB yields could probably be gained by testing new genes and targeting acetyl-CoA related pathways, in particular acetate and ethanol (Woolston et al., 2018; Lo et al., 2020). *In vitro* testing has recently proven successful in screening multiple 3HB genes (Karim et al., 2020). Furthermore, different 3HB pathways could potentially be combined in a single strain, as they do not appear to be incompatible and may improve 3HB yield/rate/titer. For instance, the *hbd1* pathway in *C. autoethanogenum* generates significant amounts of acetate, which could be reassimilated when combined with *ctfAB*/*hbd2* (Karim et al., 2020). Additionally, while Karim et al. reported impressive titers of 3HB production based on the *C. kluyveri* Hbd1 in *C. autoethanogenum*, we were unable to repeat those results in *C. ljungdahlii*, suggesting there may be strain specific differences contributing to 3HB production. It is worth emphasizing that *C. kluyveri* Hbd2 is NADH-linked, while *C. kluyveri* Hbd1 is NADPH-linked (Wang et al., 2010). NAD(P)H redox differences may be a factor in 3HB production. Improved *Clostridia* product formation is often driven by changes to NAD(P)H metabolism (Lo et al., 2017, 2015; Nguyen et al., 2018; de Souza Pinto Lemgruber et al., 2019; Karim et al., 2020; Soucaille et al., 2020). Beyond 3HB, Hbd2 could be important for other related products including PHB and longer chain fatty acids/alcohols (C4-C6) (Phillips et al., 2015; Richter et al., 2016). Hbd2 has been drastically understudied in the context of both *Clostridial* physiology and engineering, and we have shown that it could have direct biotechnological uses.

## Conclusions

To our knowledge, this is the first time *hbd2* has been utilized *in vivo* for enhancing targeted product formation. Little is known about the native function of these genes, their biochemical characteristics/activity, and how they may be utilized to improve target product formation. The native activity of Hbd2 could be unknowingly contributing to efforts to engineer 3HB/PHB/fatty acid/alcohol production in *Clostridia*, as *hbd2* is commonly found in many *Clostridia* studied for metabolic engineering. Our data shows that these Hbd2 enzymes may be a useful avenue of further interest for 3HB/PHB production. Additionally, Hbd2 catalyzes an important step in butanol/butyrate production, which are other value-added chemicals of interest that have been produced in acetogens. Furthermore, CoA-transferase based formation of 3HB appears to be a fruitful area of research for high 3HB production, especially considering that acetate formation is important for ATP synthesis in acetogens. This work sets the stage to explore the applications and diversity of *hbd2* and CoA-transferases. We anticipate that higher titers could be reached by exploring metabolic engineering strategies with these enzymes.

## Data availability statement

The original contributions presented in this study are included in the article/Supplementary material, further inquiries can be directed to the corresponding author.

## Author contributions

JL, JH, LM, SH, WX, KC, and PCM designed research. JL, JH, LM, CU, SH, and BW performed research. JL, JH, WX, KC, and PCM wrote the manuscript. All authors contributed to the article and approved the submitted version.

## Author disclaimer

The views expressed in the article do not necessarily represent the views of the DOE or the US Government. The US Government retains and the publisher, by accepting the article for publication, acknowledges that the US Government retains a nonexclusive, paid-up, irrevocable, worldwide license to publish or reproduce the published form of this work, or allow others to do so, for US Government purposes.
